# Supplementary figures and images for: The role of pneumococcal extracellular vesicles on the pathophysiology of the kidney disease hemolytic uremic syndrome
Source: mSphere. 2023 Jun 26;8(4):e00142-23. doi: 10.1128/msphere.00142-23 (PMC10449520; doi:10.1128/msphere.00142-23)

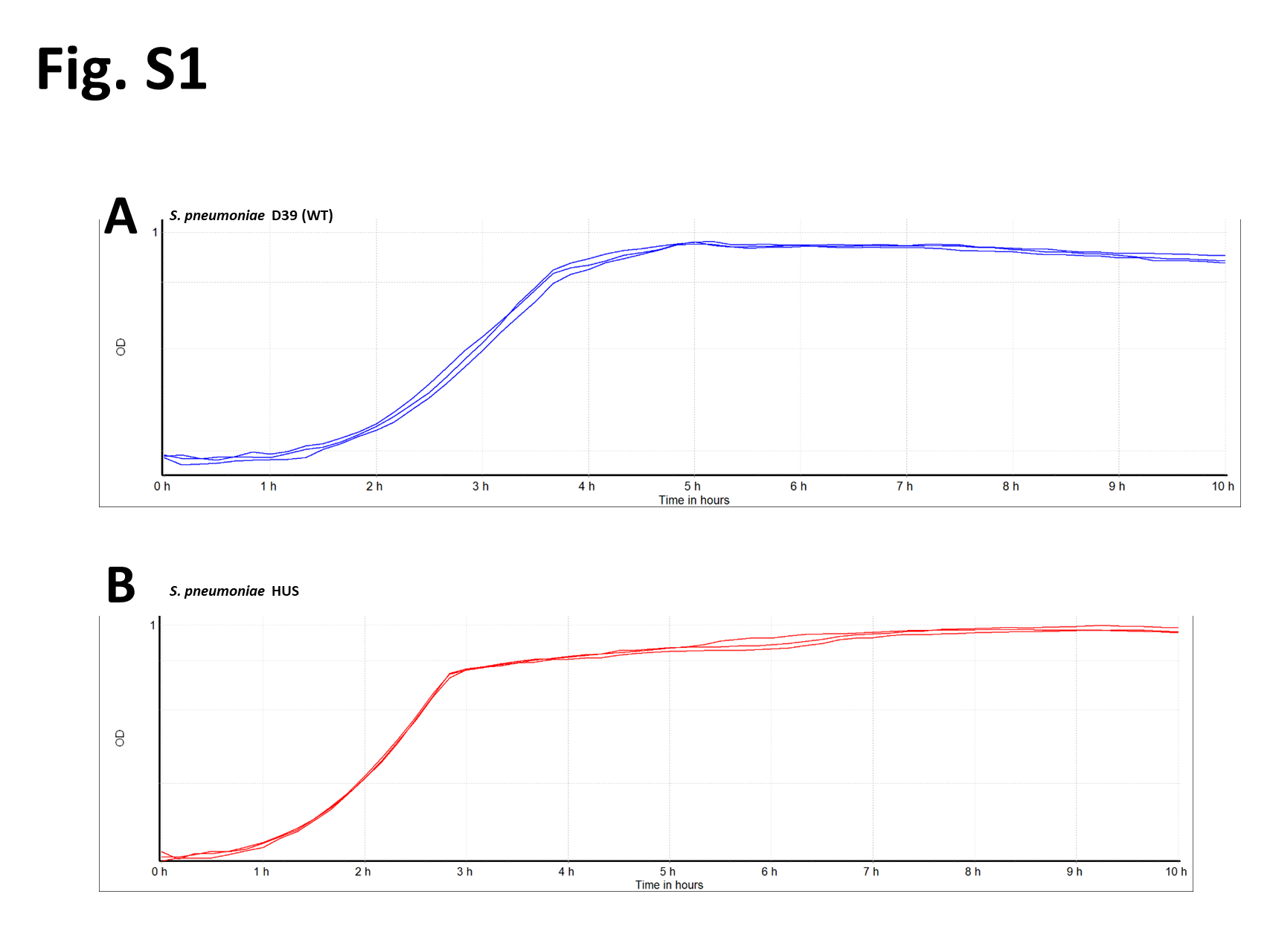

Supplement: Fig. S1 — Growth profile of WT (A) and Sp-HUS strain (B) in rich medium. [file msphere.00142-23-s0001.tif]

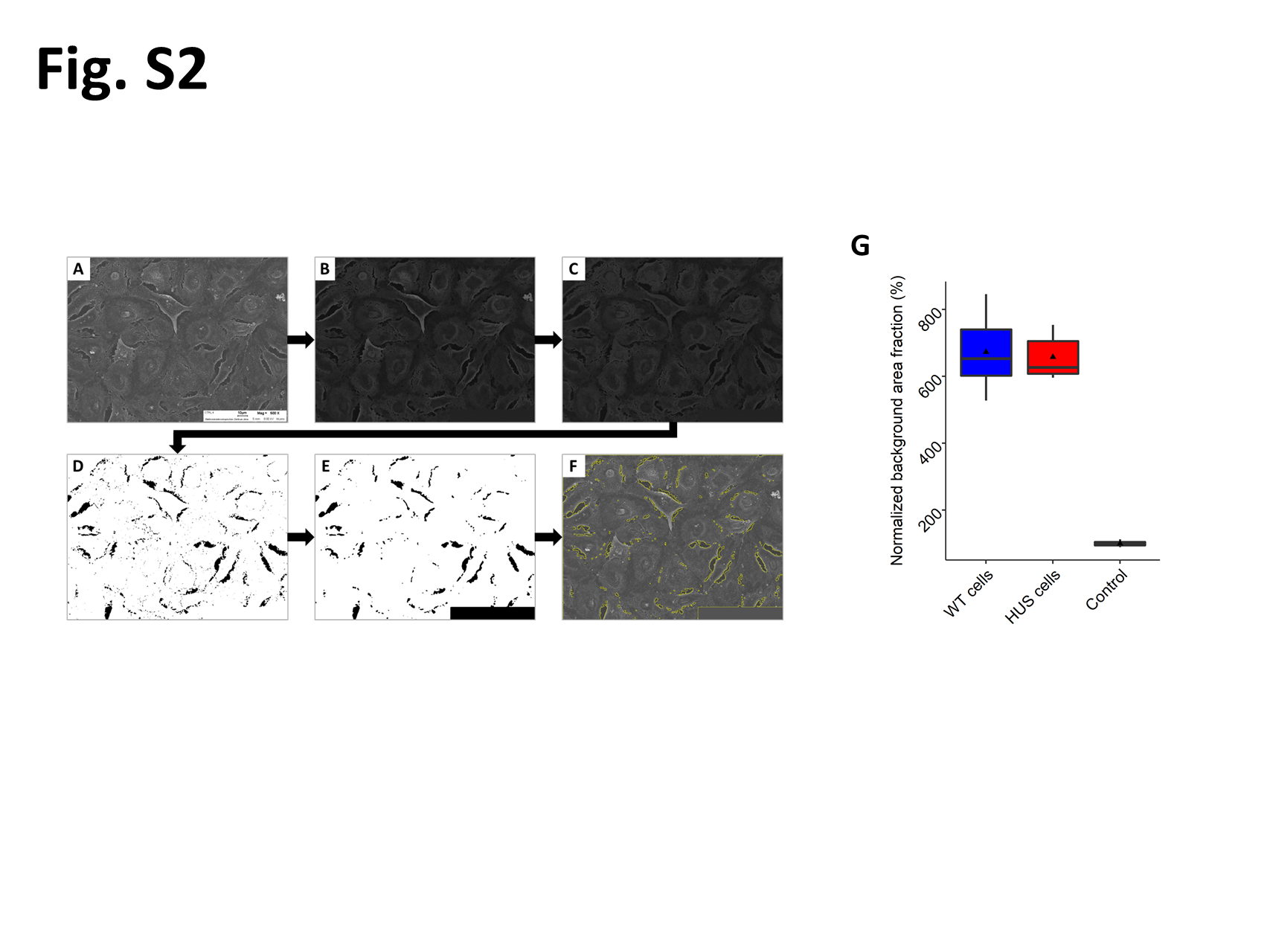

Supplement: Fig. S2 — SEM image analysis pipeline on cell retraction. [file msphere.00142-23-s0002.tif]

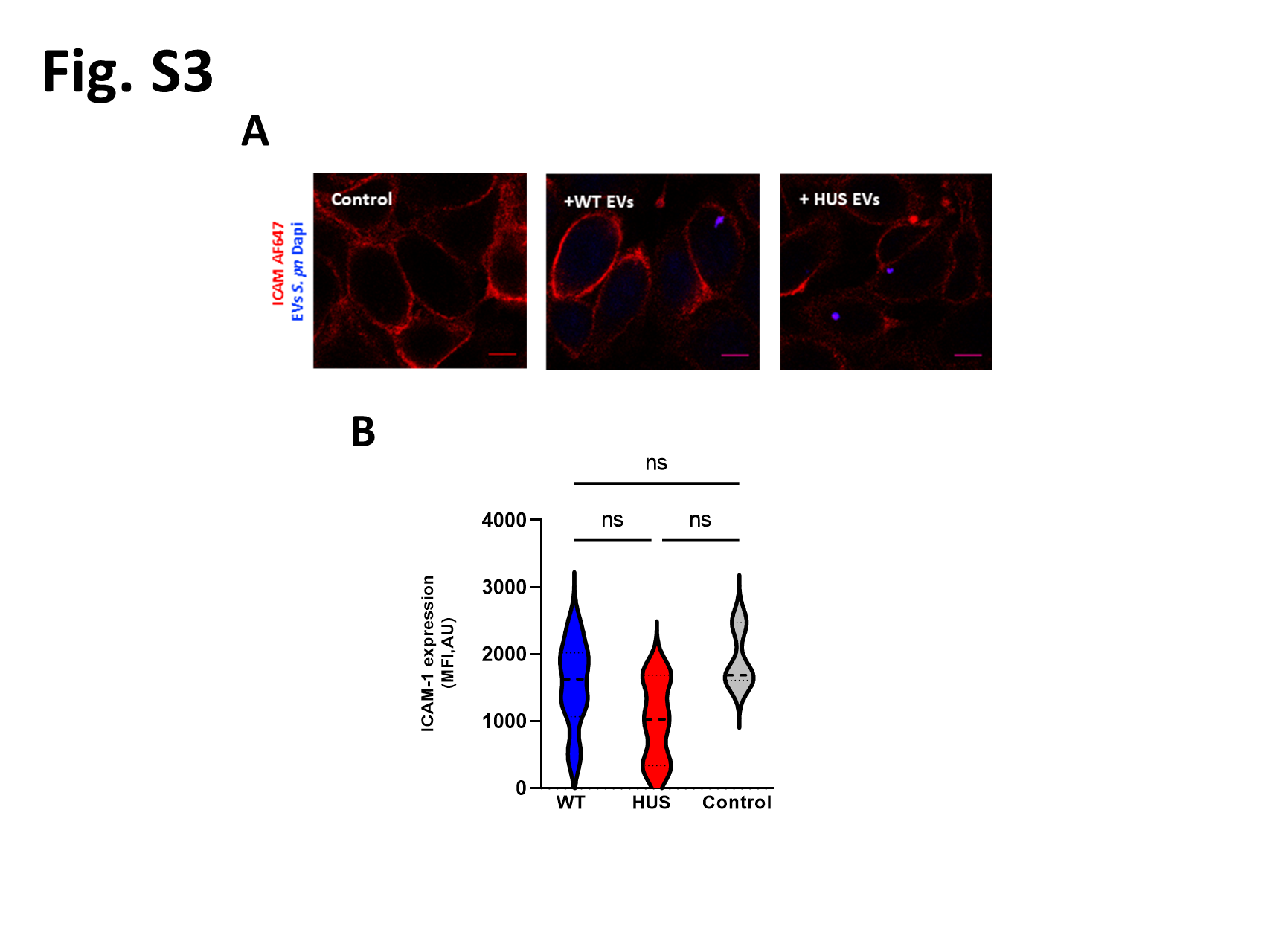

Supplement: Fig. S3 — Visualization of endothelial cells interaction with WT or Sp-HUS EVs and ICAM-1 expression by confocal-laser scanning microscopy. [file msphere.00142-23-s0003.tif]

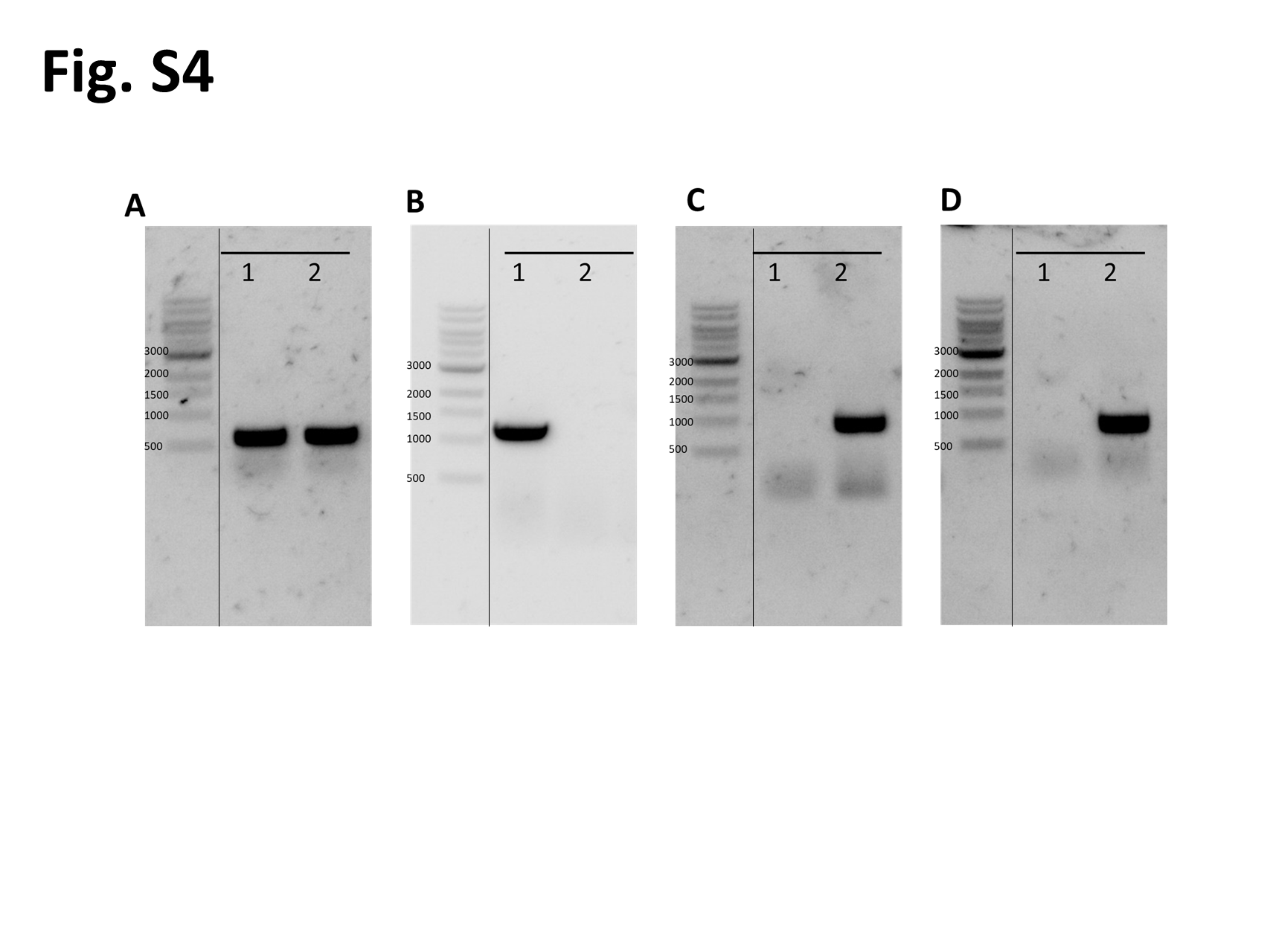

Supplement: Fig. S4 — Analysis of serotype group-specific PCR products. [file msphere.00142-23-s0004.tif]

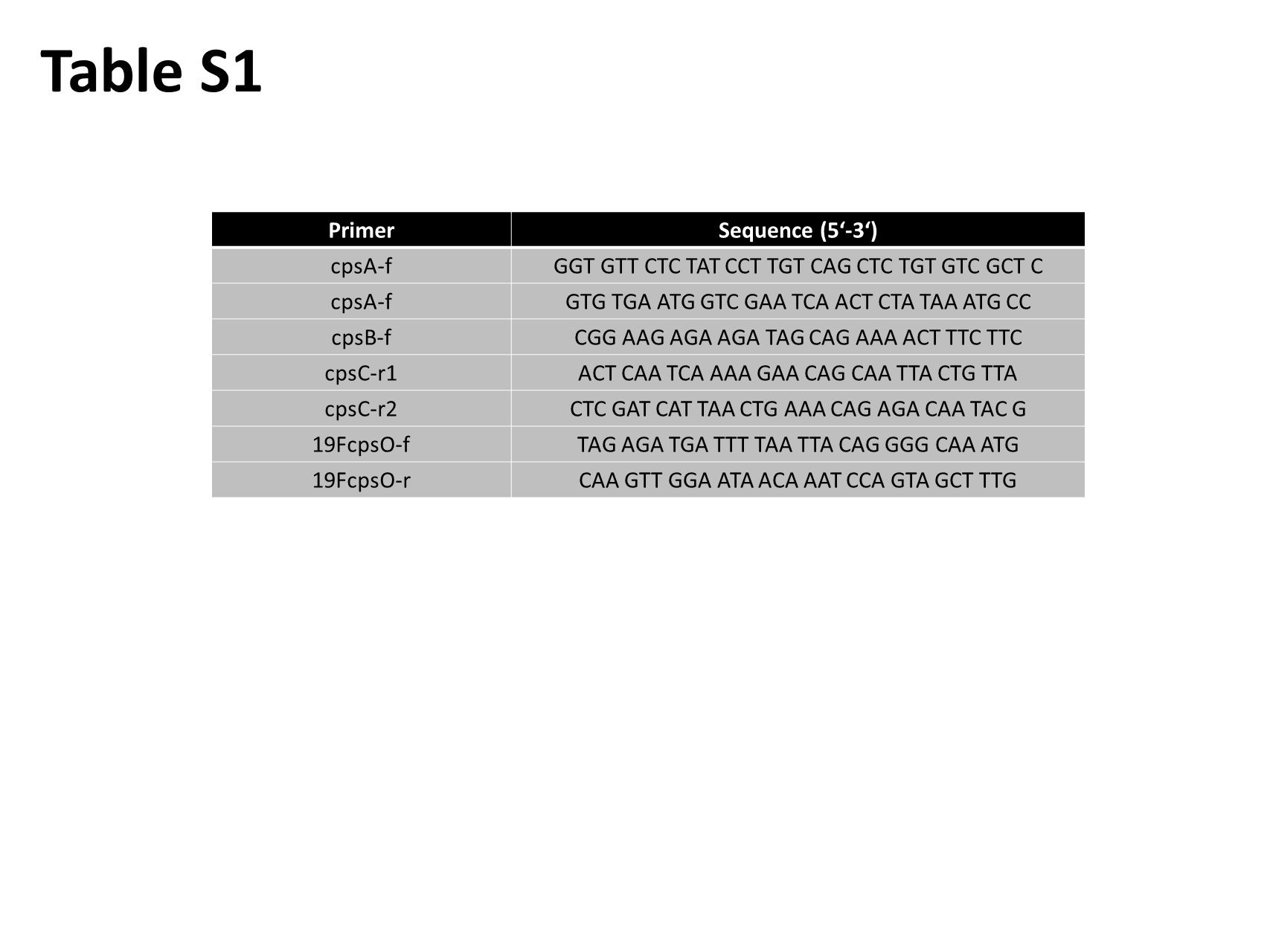

Supplement: Table S1 — Primer sequences for PCR-based serotyping. [file msphere.00142-23-s0005.tif]

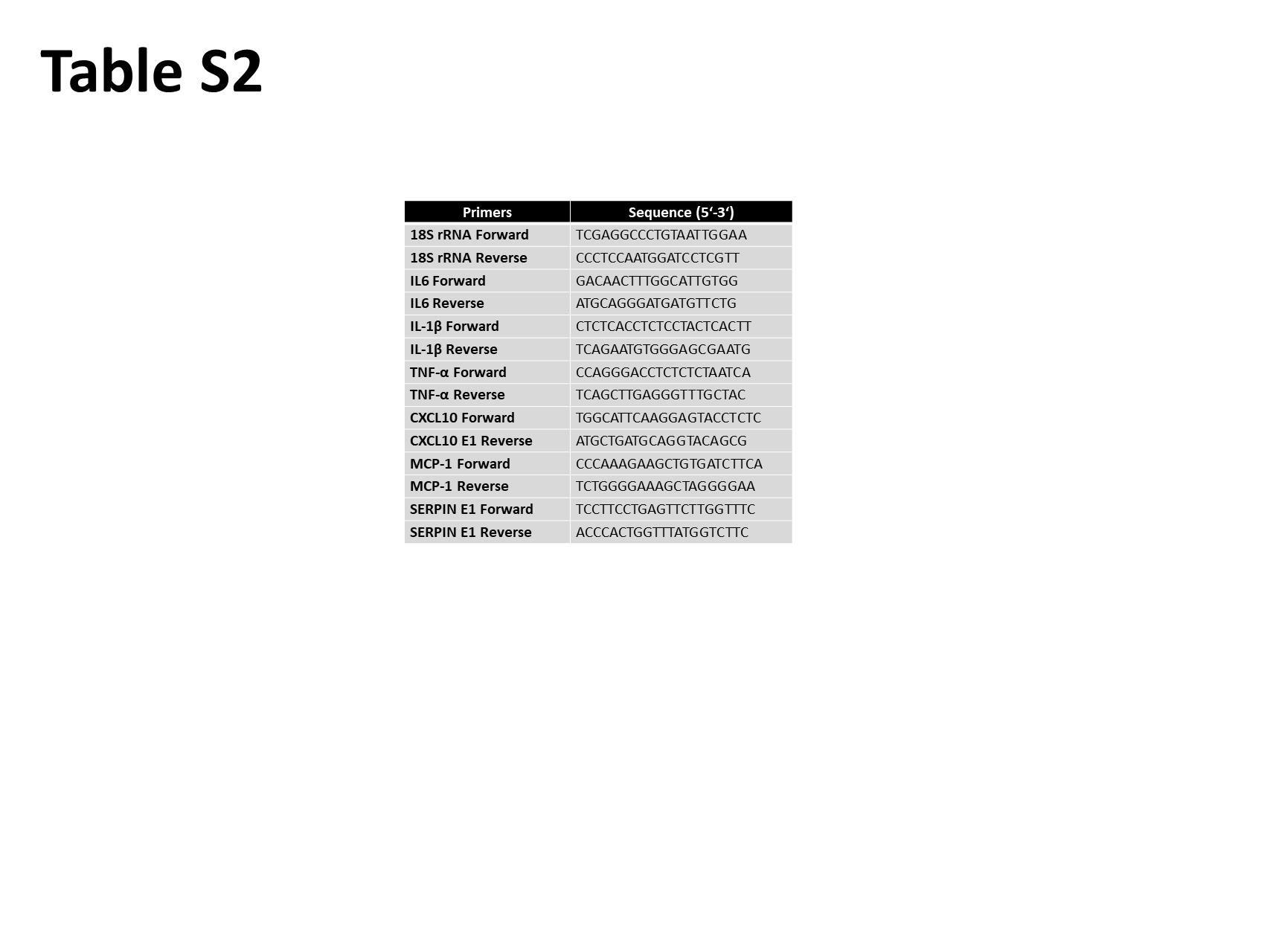

Supplement: Table S2 — Primer sequences of qPCR. [file msphere.00142-23-s0006.tif]
