# Supplementary material for: The role of pneumococcal extracellular vesicles on the pathophysiology of the kidney disease hemolytic uremic syndrome
Source: mSphere. 2023 Jun 26;8(4):e00142-23. doi: 10.1128/msphere.00142-23 (PMC10449520; doi:10.1128/msphere.00142-23)
Supplement: Supplemental File 1 — Supplemental Figure Legends and Material and Methods. [file msphere.00142-23-s0007.docx]

**SUPPLEMENTARY MATERIAL**

**The role of pneumococcal extracellular vesicles on the pathophysiology of the kidney disease Hemolytic Uremic Syndrome**

Miriana Battista^1^, Bianca Hoffmann^2^, Yann Bachelot^2^, Lioba Zimmermann^1^, Laura Teuber^1^, Aurélie Jost^3^, Susanne Linde^4^, Martin Westermann^4^, Mario M. Müller^5^, Hortense Slevogt^5^, Sven Hammerschmidt^6^, Marc Thilo Figge^2,7^, Cláudia Vilhena^1*†^, & Peter F. Zipfel^1,7*†^

^1^Department of Infection Biology, Leibniz Institute for Natural Product Research and Infection Biology, Jena, Germany

^2^Applied Systems Biology, HKI-Center for Systems Biology of Infection, Leibniz Institute for Natural Product Research and Infection Biology, Hans Knöll Institute (HKI), Jena, Germany

^3^Microverse Imaging Center, Cluster of Excellence “Balance of the Microverse”, Friedrich-Schiller-University Jena, Germany

^4^Centre for Electron Microscopy, Jena University Hospital, Jena, Germany

^5^Septomics Research Center, Jena University Hospital, Jena, Germany

^6^Department of Molecular Genetics and Infection Biology, Interfaculty Institute for Genetics and Functional Genomics, Center for Functional Genomics of Microbes, University of Greifswald, Greifswald, Germany

^7^Institute of Microbiology, Faculty of Biological Sciences, Friedrich-Schiller-University, Jena, Germany

*Authors contributed equally to this work.

^†^ **Corresponding authors:**

[Peter.zipfel@leibniz-hki.de](mailto:Peter.zipfel@leibniz-hki.de) and [Claudia.vilhena@leibniz-hki.de](mailto:Claudia.vilhena@leibniz-hki.de)
Department of Infection Biology

Leibniz Institute for Natural Products and Infection Biology – Hans Knöll Institute

Beutenbergstr 11a

07745 Jena

Germany

**Running Title:** Pneumococcal extracellular vesicles and host interaction

**Keywords:** extracellular vesicles, immunomodulation, cytokines, microbe-host

**Supplementary File 1**

**Supplementary Figure legends**

**Figure S1- Growth profile of WT (A) and Sp-HUS strain (B) in rich medium.** Cells were grown for 10h at 37°C. Optical density (OD) at 600 nm wavelength was recorded every 10 min and technical replicates were plotted.

**Figure S2- SEM image analysis pipeline on cell retraction. (A)** Exemplary original SEM image. **(B)** Images were pre-processed to reduce noise and homogenize illumination. **(C)** Images with overall low contrast or protruding bright areas were additionally processed by contrast enhancement and replacement of bright areas with lower intensity values, respectively. **(D)** Binarization into foreground (cell) and background regions was performed using a global threshold. **(E)** Bright and dark noise was removed from the binarized images. **(F)** The final segmentation of the exemplary SEM image is shown as yellow overlay. **(G)** WT and Sp-HUS strains led to increased levels of cell retraction compared to the control, while there was no significant difference between the two pathogenic strains (background area fraction of control set to 100%).

**Figure S3- Visualization of endothelial cells interaction with WT or Sp-HUS EVs and ICAM-1 expression by confocal-laser scanning microscopy.** (**A**) Confluent HUVECs were incubated with DAPI pre-stained EVs (blue), then cells were stained with a AlexaFluor647-labelled anti-ICAM-1 antibody (red). HUVECs in growth medium (DMEM) were considered the negative control. Scale bar = 10 μm. (**B**) Violin plots of the fluorescence intensity of ICAM-1 signal (red) expressed in arbitrary units (AU), measured after imaging of WT or Sp-HUS EVs and HUVECs co-incubation.

**Figure S4**- **Analysis of serotype group-specific PCR products.** Serotype group-specific genes of two *S. pneumoniae* strains (1- Sp-HUS strain, 2- WT strain) were amplified with four different primer pairs by means of PCR. (**A)** Reaction with primer pair Control; fragment size is 657 bp. (**B)** Reaction with primer pair A; fragment size 1187 bp. (**C**) Reaction with primer pair B; fragment size 980 bp. (**D**) Reaction with primer pair C; fragment size 814 bp. DNA ladder is 1 Kb.

**Table S1 – Primer sequences for PCR-based serotyping**

**Table S2- Primer sequences of qPCR**

**Supplementary File 1 – Supplementary Figure Legends and Material&Methods**

**Supplementary File 2 – List of proteins found on Sp-EVs**

**Material and Methods**

**Scanning Electron Microscopy (SEM)**

For SEM, human cells were grown on 12-well plates containing 12 mm ∅ coverslips (Roth^®^), until confluency. Bacteria were grown as described above, and co-incubation of cells and bacteria was performed for 1 hour. Then cells were fixed for 1 hour in 2.5% glutaraldehyde in sodium cacodylate buffer (0.1 M, pH 7.0) and washed three times with sodium cacodylate buffer for 20 minutes each. Samples were dehydrated in rising ethanol concentrations followed by critical point drying, using a Leica EM CPD300 Automated Critical Point Dryer (Leica) and finally coated with gold (25 nm) in a Safematic CCU-010 HV Sputter Coating System (Safematic). SEM images were acquired at different magnifications in a Zeiss-LEO 1530 Gemini field-emission scanning electron microscope (Carl Zeiss) at 6-8kV acceleration voltage and a working distance of 5-7 mm using an InLense secondary electron detector for secondary electron imaging.

All SEM images were processed with the image analysis software JIPipe v1.75.2^1^ to analyze cell retraction based on the quantification of cell and background areas (**Fig. S2A-F**). First, the scale bar region of ESM images was filled with the modal gray value of the remaining image area. Afterwards, noise was reduced with the despeckle function. Bright areas within cells were removed by gray scale attribute filtering using the operation opening with the attribute box diagonal set to a minimum value of 50 pixels and the connectivity set to 8-connected. The minimum gray value of the overall image was then subtracted from the gray value at each pixel position to ensure the comparability of intensity values across images. To separate the images into cell and background areas, an intensity threshold t_min_ was calculated using the Minimum method. In cases where no threshold could be determined due to too low contrast between cells and background, the contrast was enhanced with saturation set to 0.35 and normalization turned on, and the threshold calculation was repeated. As some images still exhibited bright regions within cells, which would lead to too large values of t_min_, the intensity of such regions was reduced. This correction was performed for images with t_min_ > 60 and the intensity of all pixel positions with

$g\left( x,y \right)\geq m_{I}+2{sd}_{I}$ *(1)*

was set to

$g^{'}\left( x,y \right)= m_{I}+{sd}_{I}$, *(2)*

where *g(x,y)* is the gray value at pixel position *(x,y)*, *m_I_* and *sd_I_* are the modal gray value and standard deviation of the entire image, and *g’(x,y)* is the new gray value assigned to the pixel position *(x,y)*. For the final segmentation, a second threshold t_Renyi_ was calculated using the Renyi entropy method and used in cases where t_Renyi_ was lower than t_min_. The pixel values in the scale bar area were set to 0 after applying the threshold to ensure that this region is always classified as background. Finally, small artifacts were removed from the binarized images by two times applying the remove outliers function with the radius set to 3, the threshold set to 50 and the target once set to dark and once set to bright areas. The background area and the area covered by cells were then quantified for each image.

**Co-incubation model of *S. pneumoniae* and HUVEC cells**

For extracellular vesicles generated from both *S. pneumoniae* and HUVECs cells (Mix-EVs), a co-incubation model was established using the Transwell® system. In short, HUVECs were seeded on the bottom of the basolateral side and let to grow at 37°C with 5% CO_2_ until confluency reached. Mid-exponentially grown *S. pneumoniae* strains were inoculated on the apical side of the inlet containing a 0,40 µm pore membrane, and the system was incubated for 24 hours. The supernatant from the basolateral side (containing both *S. pneumoniae* and HUVEC released EVs) was take and processed the same way as the above-mentioned bacterial supernatant.

**RNAseq data analysis**

The read counts files obtained from the sequenced data were used to proceed with the different analyses described below.

First, a differential gene expression analysis was performed using the package *DESeq2*. The raw read counts were loaded in R and the analysis was run using the default parameters. The fit of the model was verified by plotting the gene dispersion against the mean of the normalized counts. Genes with FC >1.5 and pval adjusted < 0.05 were considered differentially expressed (DEGs) between the two conditions.

A Gene Ontology (GO) enrichment analysis was applied to the DEGs (both up and down-regulated genes), using *Panther 17.0*^2^. The processes enriched are presented in Figure S3.

Focusing on genes sharing similar expression patterns, a co-expression network was built by applying a Pearson correlation on the genes as well as a gaussian kernel. Then, clustering using k-means was performed, and a GO enrichment on the resulting clusters. The analysis was also conducted using *Weighted Gene Coexpression Network Analysis* R package (WGCNA^3^), both approaches giving similar results. The obtained network was then imported into *Cytoscape*^4^. for further manipulations. The network is presented in Figure S3.

**Serotyping by multiplex PCR**

PCR was performed to identify the capsular serotype of Sp-HUS strain and one reference strain with known serotype (WT). The protocol was adapted from a multiplex PCR protocol created by Brito et al.^5^. In this protocol, *S. pneumoniae* strains are first divided into one of six possible groups by evaluating the products of a group PCR reaction and afterwards, a specific reaction determines the serotype. Genomic DNA (gDNA) was isolated from the *S. pneumoniae* strains with the Qiagen DNeasy® Blood & Tissue Kit according to the manufacturer’s instructions. The concentration and purity of the DNA were confirmed by NanoDrop-1000 Spectrophotometer. The primer combinations used for the group reaction and the expected fragment size were: Control: cpsA-f/cpsA-r (657 bp), cpsB-f/cpsC-r1 (1,187 bp), cpsB-f/cpsC-r2 (980 bp) and 19FcpsO-f/19FcpsO-r (814 bp).. Primer sequences are showed in **Table S1**. Primer sets B, C and D were designed by Brito et al. to amplify specific regions of genes which are common to groups of serotypes. Primer pair A served as an internal control for all targeted serotypes to confirm suitability of gDNA. Instead of one multiplex group reaction, PCR protocols were adapted and performed individually for each primer set, considering the primer annealing temperature and the resulting fragment length. The resulting PCR products were subsequently analyzed by electrophoresis in 1% (w/v) agarose gels (in 1x Tris base, acetic acid and EDTA, TAE buffer). Gels were run at 120 V for approximately 45 min. Gel imaging was performed with the Syngene G:Box.

**Supplementary References:**

1. Gerst, R., Cseresnyés, Z. & Figge, M. T. JIPipe: visual batch processing for ImageJ. *Nat Methods* (2023) doi:10.1038/s41592-022-01744-4.

2. Thomas, P. D. *et al.* PANTHER: A library of protein families and subfamilies indexed by function. *Genome Res* **13**, (2003).

3. Langfelder, P. & Horvath, S. WGCNA: An R package for weighted correlation network analysis. *BMC Bioinformatics* **9**, (2008).

4. Shannon, P. *et al.* Cytoscape: A software Environment for integrated models of biomolecular interaction networks. *Genome Res* **13**, (2003).

5. Brito, D. A., Ramirez, M. & de Lencastre, H. Serotyping Streptococcus pneumoniae by multiplex PCR. *J Clin Microbiol* **41**, (2003).
